# Supplementary material for: Regional analysis of spatiotemporal trends in Colorado potato beetle abundance
Source: Pest Manag Sci. 2026 Jul 6;82(12):11162–71. doi: 10.1002/ps.71109 (PMC13756823; doi:10.1002/ps.71109)
Supplement: Supplementary file 1 [file PS-82-11162-s001.docx]

**Supporting Information**

**A**

**B**

**Figure S1**. Variation in A) adult and B) larval Colorado potato beetle abundance in Wisconsin potato fields for entire dataset.

**Figure S2**. Variation in adult and larval Colorado potato beetle abundance, as well as measures of potato proportion and potato intensity, in Wisconsin potato fields through time. Weighted potato proportion represents the amount of potato grown at a field within a five-year period and weighted intensity describes the total area of potato grown within a 1.5km radius of a field centroid over a five-year period.

**Figure S3**. Distribution of values for response and predictor variables.

**Figure S4**. Cross-correlation of predictor variables. Variables were considered for removal if any pairwise correlation was |r|>0.7.

|  |  |  |
| --- | --- | --- |

**Figure S5**. Generalized additive model (GAM) diagnostics. Partial effects showing the spatial smoother and the random-effect smoothers for year and farm.

**Figure S6**. Residual diagnostics from the spaMM model, including quantile–quantile plots, residual vs. fitted scatterplots, and residuals over space.

**Figure S7**. Scenario maps from the spaMM model showing the statewide CPB risk surface when held at (a) baseline, (b) the landscape potato intensity within 1.5 km is reduced by 30%, (c) the landscape potato intensity within 1.5 km is increased by 50%, (d) there are more (2-fold) heavier summer rain events, (e) winters are warmer by 2^o^C, and (f) summer cumulative growing degrees are warmer by 10%. The other predictors fixed in each scenario as in the baseline map; year fixed to 2021; farm random effects omitted for out-of-sample generalization. Risk rescaled to a 0–1 index.

**Table S1.** Summary statistics of values for response and predictor variables.

|  | cpb_adult | cpb_larvae | tmin | tmax | gdd | cum_gdd | intensity | potato_proportion | tmin_annual | tmax_annual | annual_prcp | summer_tmin | summer_avg | summer_prcp | winter_avg | winter_tmax |
| --- | --- | --- | --- | --- | --- | --- | --- | --- | --- | --- | --- | --- | --- | --- | --- | --- |
| observations | 4287 | 4287 | 3040 | 3040 | 3040 | 3040 | 4287 | 4287 | 4287 | 4287 | 4287 | 4287 | 4287 | 4287 | 4287 | 4287 |
| mean value | 2.538 | 4.226 | 46.724 | 89.193 | 16.736 | 1709.851 | 0.144 | 0.345 | -28.275 | 34.448 | 3.144 | -1.195 | 19.256 | 3.397 | -6.096 | 12.494 |
| std | 2.495 | 3.117 | 5.067 | 3.389 | 1.765 | 273.342 | 0.098 | 0.144 | 2.911 | 1.873 | 4.867 | 2.570 | 0.892 | 3.940 | 1.946 | 3.947 |
| min | 0.000 | 0.000 | 20.334 | 33.959 | 0.000 | 0.000 | 0.000 | 0.000 | -37.800 | 29.900 | 1.004 | -12.000 | 16.453 | 0.812 | -13.488 | 4.800 |
| 25% | 1.133 | 2.200 | 44.180 | 87.389 | 15.674 | 1595.082 | 0.066 | 0.200 | -30.600 | 33.000 | 1.389 | -2.800 | 18.807 | 1.848 | -7.227 | 9.400 |
| 50% | 2.107 | 3.750 | 46.904 | 89.156 | 16.822 | 1738.222 | 0.127 | 0.400 | -27.200 | 34.400 | 1.739 | -1.600 | 19.348 | 2.288 | -6.418 | 12.200 |
| 75% | 3.426 | 5.678 | 50.272 | 91.193 | 17.867 | 1882.117 | 0.205 | 0.400 | -26.700 | 35.300 | 2.320 | 0.300 | 19.689 | 3.017 | -4.423 | 15.600 |
| max | 98.727 | 46.333 | 64.416 | 101.402 | 25.226 | 2296.555 | 0.578 | 0.800 | -21.600 | 38.900 | 31.200 | 6.100 | 21.221 | 23.600 | -2.517 | 21.000 |

**Table S2**. Likelihood ratio tests (LRTs) for random effects in linear mixed-effects model (LMM).

| **Model comparison** | **df** | **AIC (logLik)** | **Chisq (p-value)** |
| --- | --- | --- | --- |
| Null (intercept only) vs. + Farm RE | 1 | 12525  (-6259.4) | 378.18 ($<2.2\times{10}^{-16})$ |
| Farm RE vs. + Year RE | 1 | 11980  (-5985.9) | 546.92 ($<2.2\times{10}^{-16})$ |

**Table S3**. Fixed effect estimates (with 95% Wald confidence intervals) from the linear mixed-effects model (LMM). Fixed effect estimates with t values exceeding 1.96 and whose confidence intervals did not overlap zero were considered significant and labeled with an asterisk. GDD represents growing degree days, while weighted potato proportion represents the amount of potato grown at a field within a five-year period, and weighted intensity describes the total area of potato grown within a 1.5km radius of a field centroid over a five-year period. Both measures are weighted towards more recent years.

| **Term** | **Estimate (Std. Error)** | $\boldsymbol{t}$ **value [95% CI]** |
| --- | --- | --- |
| Intercept | 3.506 (0.279) | 12.58 [2.960, 4.052] |
| GDD | -0.001 (0.033) | -0.04 [-0.065, 0.062] |
| Cumulative GDD | 0.085 (0.031) | 2.70 [0.023, 0.147]* |
| Weighted Intensity | 0.286 (0.034) | 8.38 [0.219, 0.353]* |
| Weighted Proportion | 0.053 (0.028) | 1.89 [-0.002, 0.109] |
| Summer Avg. Temp | -0.075 (0.063) | -1.19 [-0.198, 0.048] |
| Summer Avg. Precipitation | -0.027 (0.032) | -0.84 [-0.091, 0.036] |
| Heavy Rainfall Days | -0.029 (0.038) | -0.76 [-0.103, 0.045] |
| Hottest Summer Temp | 0.033 (0.062) | 0.53 [-0.089, 0.155] |
| Coldest Winter Temp | 0.082 (0.067) | 1.22 [-0.049, 0.213] |
| Winter Extreme Cold Days | 0.026 (0.057) | 0.45 [-0.086, 0.138] |
| Winter Warm Day Count | -0.059 (0.058) | -1.02 [-0.172, 0.054] |
